# Toscana virus (TOSV) meningitis with atypical characteristics: Report of two cases

**DOI:** 10.1016/j.idcr.2024.e02034

**Published:** 2024-07-21

**Authors:** Roberta Maria Antonello, Giuseppe Formica, Letizia Attala, Dario Mannini, Lorenzo Zammarchi, Alessandro Bartoloni, Massimo Antonio Di Pietro

**Affiliations:** a Department of Experimental and Clinical Medicine, University of Florence, Florence, Italy; b Infectious Diseases Unit, Ospedale Santa Maria Annunziata, Bagno a Ripoli, Florence, Italy; c Infectious and Tropical Diseases Unit, Careggi University Hospital, Florence, Italy

**Keywords:** CNS infection, Emerging infectious diseases, *Phlebovirus*, Vector borne diseases

## Abstract

Toscana virus (TOSV) is an emerging cause of central nervous system (CNS) infections, especially in endemic countries during summer. Cerebrospinal fluid (CSF) is usually clear, with < 500 leukocytes/mm^3^, normal glucose (> 60 % serum glucose) and normal (< 45 mg/dL) to slightly increased protein levels. Here we present two cases of TOSV meningitis with misleading CSF characteristics observed at Santa Maria Annunziata Hospital (Bagno a Ripoli, Florence, Italy). Case 1 presented with signs and symptoms of meningitis. CSF was opalescent on macroscopic examination, with 1192 cells/mm^3^, hypoglycorrhachia (30 % serum glucose) and hyperproteinorachia (228.0 mg/dL). TOSV meningitis was confirmed with serology. Case 2 presented with headache, vomiting and mild neck stiffness. CSF was slightly turbid, with 1092 cells/mm^3^, normal glucose (61 % serum glucose) and slightly increased protein (77.0 mg/dL) levels. TOSV meningitis was confirmed with serology and molecular test on CSF. We performed a literature review including cases of TOSV neuroinvasive infections in which CSF characteristics were reported. Pleocytosis > 500 cells/mm^3^ was reported in 12/62 (19.4 %) patients, hypoglycorrhachia in 3/62 (4.8 %) patients, mild hyperproteinorachia (45 - 75 mg/dL) in 7/62 (11.3 %) patients and severe hyperproteinorachia (> 75 mg/dL) in 40/62 (64.5 %) patients. TOSV should be considered in the differential diagnosis of CNS infections in endemic areas during the warm season even when CSF examination shows atypical results.

## Highlights

• TOSV is an emerging cause of central nervous system infections.
• We report two cases of TOSV meningitis with atypical CSF characteristics.
• We performed a literature review to report frequency of atypical findings.
• A prompt diagnosis avoids antibiotics and prolonged inhospital stay.
• Climate change is extending the areas of activity of phlebotomine vectors.

## Introduction

Among pathogens responsible for CNS infections, Toscana virus (TOSV) is emerging in endemic regions during the warm season (most cases in the period July - September) [1], [2]. First isolated in 1971 from *Phlebotomus perniciosus* and *Phlebotomus perfiliewi* in Tuscany, TOSV is an RNA virus belonging to the genus *Phlebovirus*, family *Phenuiviridae*, transmitted to humans by sand flies of the genus *Phlebotomus* [1], [3]. It is endemic in the Mediterranean basin with epidemiological studies reporting human seroprevalence of 5.3–45 % in rural areas [4], [5], [6], [7]. Young men are more affected than women or older individuals as they are generally more involved in occupational outdoor activities [1], [2]. Three different lineages (A-C) were described so far with different geographical distributions, with lineage A being the most common in Italy, France, Tunisia, lineage B in Spain, Portugal, France, Morocco, Croatia and lineage C in Croatia and Greece [8], [9], [10], [11].

Most human infections run asymptomatic or with mild aspecific symptoms, but TOSV has the potential to cause systemic signs of infection, with symptoms ranging from flu-like syndrome to central or peripheral nervous system irritative syndromes. Other site involvement, such as the urogenital tract, is reported occasionally [12]. Neuroinvasive infections generally occur after an incubation period of 7–15 days (sporadic cases up to 21 days). Although neurological complications or sequelae and exitus have been reported occasionally, due to underdiagnosis the real disease burden is extremely difficult to estimate [11], [13], [14], [15].

The diagnosis of TOSV neuroinvasive infection is based on clinical presentation and detection of TOSV RNA on CSF, TOSV IgM on serum, or demonstration of seroconversion [16]. Blood tests and physical examination of CSF are complementary diagnostic tools. TOSV CNS infections are expected as acute clear fluid meningitis/meningoencephalitis with physical examination similar to those of other viral infections (normal glucose and slightly increased protein levels) [9].

Here we describe two cases of TOSV meningitis presenting with misleading CSF physical characteristics observed at Santa Maria Annunziata Hospital (Bagno a Ripoli, Florence, Italy).

## Case report

Case 1 A 35-year-old man with no past medical history presented to the emergency department in July 2023 with a 24-hour history of fever, vomit, frontal headache and myalgia. He lived in a rural area of Tuscany (Italy) and he reported gardening ten days prior to presentation.

On physical examination, the patient was alert (Glasgow Coma Scale 15), febrile (temperature 38.6 °C), with peripheral oxygenation 97 % in room air and blood pressure 110/70 mmHg. Mild neck stiffness and positive left Lasegue sign were reported. No neurological deficits or cutaneous rash were observed. Blood tests showed normal white blood cells (WBC) count, C reactive protein (CRP) 1.08 mg/dL (reference value < 0.5 mg/dL), procalcitonin 0.03 ng/mL (reference value < 0.5 ng/mL), fibrinogen 599 mg/dL, lactate dehydrogenases (LDH) 629 U/L, normal kidney and liver function. Brain CT scan and chest X-ray were unremarkable.

A lumbar puncture was performed, revealing a slightly turbid (“opalescent”) fluid, with 1192 cells/mm^3^ (78.4 % mononuclear), hypoglycorrhachia (29 mg/dL, 30 % of serum glucose) and hyperproteinorachia (228.0 mg/dL). A multiplex polimerase chain reaction (PCR) panel including *Escherichia coli*, *Haemophilus influenzae*, *Listeria monocytogenes*, *Neisseria meningitidis*, *Streptococcus agalactiae*, *Streptococcus pneumoniae*, *Cytomegalovirus*, *Enterovirus*, *Herpes simplex virus 1 - 2*, *Human herpesvirus 6*, *Human parechovirus*, *Varicella zoster virus*, *Cryptococcus neoformans*/*gattii* performed on CSF resulted negative. Pending culture results, the patient was started on ceftriaxone 2 g intravascular (IV) q 12 h and ampicillin 3 g IV q 6 h. Five days after collection, the CSF and blood cultures turned negative. Taking into account the local and seasonal epidemiology we performed a serology for TOSV, with detection of positive IgM (index 9.6, negative value < 0.8) and IgG (index 3.0, negative value < 0.8) (full diagnostic workup reported in Table 1), therefore antibiotics were suspended. The patient recovered well with no sequelae.

Case 2 A 18-year-old man with no past medical history presented to the emergency department in July 2023 with a 24-hour history of frontal headache and vomiting. He lived in Tuscany, and worked as a waiter in a restaurant in a rural area. On physical examination, the patient was alert (Glasgow Coma Scale 15) and afebrile. Mild neck stiffness, but no neurological deficits were described. Blood tests showed normal WBC count, CRP 2.1 mg/dL, procalcitonin 0.02 ng/mL, fibrinogen 504 mg/dL, normal kidney and liver function. A brain CT scan and a chest X-ray were unremarkable. As headache did not resolve with anti-inflammatory drugs, lumbar puncture was performed, and the physical examination showed slightly turbid fluid with 1092 cells/mm^3^ (prevalence of monocytes), with normal glucose (66.0 mg/dL, 61 % of plasma glucose) and slightly increased protein (77.0 mg/dL) levels. A multiplex PCR panel on CSF resulted negative. Ceftriaxone 2 g IV q 12 h and acyclovir 750 mg IV q 8 h were started. PCR for TOSV RNA on CSF resulted positive. The serology for TOSV turned positive for both IgM (index 2.4, negative value < 0.8) and IgG (index 4.4, negative value < 0.8) (Table 1). CSF and blood cultures turned negative. Antibiotic and antiviral treatments were discontinued and the patient was discharged home with no sequelae at follow-up.

**Table 1:** Laboratoristic findings of the two cases.

	Case 1	Case 2
CSF physical examination		
Aspect	Opalescent	Slightly turbid
Cells (n/mm^3^)	1192 (78.4 % mononuclear)	1092 (76.6 % mononuclear)
Glucose CSF/serum ratio	0.30	0.61
Proteins (mg/dL)	228.0	77.0
Lactate (mg/dL)	40.0	23.0
Blood tests (first evaluation)		
White blood cells (10^3^/μL)	7.96	9.75
Red blood cells (10^6^/μL)	5.35	5.62
Hemoglobin (g/dL)	16.6	17.4
Platelets (10^3^/μL)	167.0	278.0
C reactive protein (mg/dL, RV < 0.5 mg/dL)	1.08	2.1
Procalcitonin (ng/mL, RV < 0.5 ng/mL)	0.03	0.02
LDH (U/L)	629	221.0
Fibrinogen (mg/dL)	599	504
Creatinine (mg/dL)	0.86	0.88
ALT/AST (U/L)	48/39	21/25
Serological tests		
TOSV IgG+	Positive, index 3.0	Positive, index 4.4
TOSV IgM+	Positive, index 9.6	Positive, index 2.4
*Treponema pallidum*, *Borrelia burgdorferi*, *Mycoplasma pneumoniae*, West Nile virus	Negative	Negative
Molecular tests		
PCR TOSV RNA su liquor	Not tested	Positive
PCR multiplex (film array) meningitis panel*	Undetected	Undetected
PCR *M. tuberculosis* complex	Negative	Negative
Microscopy		
Acid fast bacilli	Negative	Negative
Cultures		
CSF cultures	Negative	Negative

ALT: alanine aminotransferase; AST: aspartate aminotransferase; CSF: cerebrospinal fluid; LDH: lactate dehydrogenase; PCR: polymerase chain reaction; RV: reference value; TOSV: Toscana virus

## Literature review

We performed a literature review on PubMed/Medline and Scopus (keywords: “TOSV”, “Toscana virus”, “CNS infection”, “meningitis”, “encephalitis”, “meningoencephalitis”) including cases of TOSV neuroinvasive infections with the purpose of bringing into context our cases looking for other atypical findings (Table S1). Cases reported from inception to 24th July 2023 were reviewed. Inclusion criteria were case report and case series with CSF physical examination, supposed country of acquisition and diagnostic tests reported. We excluded case reports for which these data were missing, as well as reviews and expert opinions. No language limitations were set.

Data on CSF physical characteristics were available for 62 patients (14 females, 34 males, 14 not specified, 19 children < 18 years, 36 individuals aged 18 - 65 years, 7 individuals > 65 years) with CNS infections (28 meningitis, 6 encephalitis, 14 meningoencephalitis, 14 not specified). Italy was the supposed country of acquisition in most cases (48/62, 77.4 %). Some cases were observed in countries different from the supposed ones of acquisition: USA (three cases), Switzerland (three cases), France (three cases), United Kingdom (two cases), Germany (two cases), Australia (one case). With regards to atypical findings of CSF, pleocytosis > 500 cells/mm^3^ was reported in 12/62 (19.4 %) patients (up to 3500 cells/mm^3^). CSF glucose ≤ 40 mg/dL was reported in 3/62 (4.8 %) patients. In another case, low CSF glucose level was reported but not specified. Mild hyperproteinorachia (45 – 75 mg/dL) was described in 7/62 (11.3 %) patients and severe hyperproteinorachia (> 75 mg/dL) in 40/62 (64.5 %) patients. Diagnostic confirmation was achieved with serology in most cases (59/62, 95.1 %).

## Discussion

We presented two cases of patients living in a highly endemic region for TOSV, presenting in July with meningitis. Moderate pleocytosis, normal or mildly elevated protein and normal glucose levels are the expected findings from CSF examination [17]. In our cases, diagnosis was challenging due to the atypical physical CSF examination, highlighting the need for awareness of emerging causative agents of CNS infections and their epidemiological features to confirm the diagnosis, estimate the real burden disease, and avoid unnecessary antibiotic courses. Atypical CSF presentation should not be considered an exclusion criteria for TOSV infection a priori, but it should be brought into the context of local epidemiology, investigating TOSV together with other neurotrophic agents. The literature review we performed suggests the need for a revision of expected CSF physical characteristics, as “unusual” features, such as non-clear optical aspect, elevated pleocytosis or hypoglycorrhachia, though rare, are described. Due to the limited number of previous papers describing atypical CSF characteristics, it was not possible to find commonalities between them and the cases we reported to suggest a possible explanation of host-virus interactions resulting in these unexpected findings. In addition, it poses relevant highlights on the epidemiology: though all cases were supposed to be acquired in the Mediterranean basin, they were observed also in non-endemic countries in returning travellers.

With this in mind, in the case of aseptic meningitis with an unclear etiology, emerging arboviruses such as phleboviruses (e.g. TOSV, sandfly fever Naples virus (SFNV), Corfu virus (CFUV), sandfly fever Sicilian virus (SFSV)) or flaviviruses (e.g. West Nile virus (WNV), Usutu virus (USUV)) should be investigated.

One limitation of the cases we presented is that TOSV RNA on CSF was not performed in Case 1 as CSF was collected in the emergency department and it was not possible to collect a separate aliquot to perform the molecular test. However, the cases included in the review alongside previous studies described a higher sensitivity of serology compared to molecular tests on CSF [2], [10], [18], [19], [20].

## Conclusion

Despite being an emerging agent of viral meningitis, TOSV remains a neglected underdiagnosed virus in CNS infections. Though not routinely investigated in individuals presenting with aseptic meningitis, TOSV should be ruled out in endemic regions especially during the period of activity of phlebotomine vectors. An atypical laboratory presentation should not be considered an exclusion criteria a priori if clinical suspicion based on epidemiology is strong. Awareness of the pathogenic role of TOSV is advisable also for clinicians working in non-endemic areas, as climate change is modifying the areas of activity of vectors and there is an increasing number of cases observed in returning travellers.

## Ethics approval and consent to participate

The study was carried out in accordance with the recommendations contained in the Declaration of Helsinki and revised at successive world assemblies. The patients provided informed consent for the publication of the cases.

## Funding

The authors declare that no funds, grants, or other support were received during the preparation of this manuscript.

## CRediT authorship contribution statement

**Alessandro Bartoloni:** Writing – review & editing, Supervision, Methodology. **Massimo Antonio Di Pietro:** Writing – review & editing, Supervision, Methodology, Conceptualization. **Roberta Maria Antonello:** Writing – original draft, Methodology, Formal analysis, Data curation, Conceptualization. **Giuseppe Formica:** Writing – original draft, Data curation, Conceptualization. **Letizia Attala:** Writing – review & editing, Formal analysis, Data curation. **Dario Mannini:** Writing – original draft, Formal analysis, Data curation. **Lorenzo Zammarchi:** Writing – review & editing, Supervision, Methodology.

## Declaration of Competing Interest

all authors declare they have no competing interests.
