# Supplementary material for: Toscana virus (TOSV) meningitis with atypical characteristics: Report of two cases
Source: IDCases. 2024 Jul 21;37:e02034. doi: 10.1016/j.idcr.2024.e02034 (PMC11318471; doi:10.1016/j.idcr.2024.e02034)
Supplement: Supplementary file 1 — Supplementary material [file mmc1.docx]

**Table S1**. TOSV neuroinvasive infections described in literature with available CSF characteristics.
CSF: cerebrospinal fluid; E: encephalitis; L: lymphocytes; M: meningitis; ME: meningoencephalitis; Mo: mononucleates; N: neutrophils; NR: not reported; NT: not tested; P: polymorphonucleates; TOSV: Toscana virus; WBC: white blood cell.

| **Ref** | **Age (years)** | **Sex at birth** | **Supposed Country of acquisition** | **Country of presentation** | **Clinical presentation** | **CSF optical aspect** | **CSF WBC count, cells/mm^3^ (type of prevalent WBC)** | **CSF glucose, mg/dL** | **CSF proteins, mg/dL** | **Serology** | **CSF PCR TOSV** | **Blood PCR TOSV** |
| --- | --- | --- | --- | --- | --- | --- | --- | --- | --- | --- | --- | --- |
| [[1]](https://paperpile.com/c/d5ULsP/t1o1) | 23 | M | Italy | Switzerland | M | NR | 53 (L) | Normal, NR | 82.1 | IgM+/IgG+ | Pos | Pos |
| [[2]](https://paperpile.com/c/d5ULsP/nNbi) | 21 | M | Croatia | Croatia | M | NR | 175 (L) | 68 | 44.7 | IgM+/IgG+ | NT | NT |
|  | 22 | F | Croatia | Croatia | ME | NR | 102 (L) | 54 | 99.3 | IgM+/IgG+ | NT | NT |
|  | 54 | M | Croatia | Croatia | M | NR | 123 (L) | 73 | 106.5 | IgM+/IgG+ | NT | NT |
| [[3]](https://paperpile.com/c/d5ULsP/fhmP) | 33 | F | Italy | Italy | ME | Clear | 330 (Mo) | 63% of serum glucose | 184 | IgM+/IgG- | Neg | NT |
| [[4]](https://paperpile.com/c/d5ULsP/csWL) | 27 | F | Tunisia | Tunisia | ME | NR | 290 (L) | Low, NR | 167 | IgM+/IgG NR | NT | Pos |
| [[5]](https://paperpile.com/c/d5ULsP/0f8U) | 44 | M | Spain | France | M | NR | 51 (N) | 68 | 94 | NT | Pos | NT |
| [[6]](https://paperpile.com/c/d5ULsP/sAcG) | 17 | F | Italy | United Kingdom | M | NR | 120 (L) | 59 | 20 | IgM+/IgG+ | Pos | NT |
| [[7]](https://paperpile.com/c/d5ULsP/ebzW) | 51 | M | Italy | Australia | M | NR | 82 (Mo) | 58 | 71 | NT | Pos | NT |
| [[8]](https://paperpile.com/c/d5ULsP/aLIJ) | 65 | M | Italy | United Kingdom | E | Turbid | 712 (L) | 68 | 159 | IgM NR/IgG+ | Pos | NT |
| [[9]](https://paperpile.com/c/d5ULsP/RsDS) | 82 | M | Italy | USA | E | NR | 86 (L) | 59 | 169 | IgM+/IgG NR | NT | NT |
| [[10]](https://paperpile.com/c/d5ULsP/QkWU) | 61 | M | Italy | Switzerland | M | NR | 134 (L) | 54 | 78 | IgM+/IgG+ | Pos | Neg |
| [[11]](https://paperpile.com/c/d5ULsP/MRMK) | 40 | F | France | France | ME | Clear | 475 (L) | 43 | 178 | IgM-/IgG- | Pos | NT |
| [[12]](https://paperpile.com/c/d5ULsP/kYnd) | 48 | M | Italy | Italy | ME | NR | 465 (L) | 51 | 200 | IgM+/IgG NR | Pos | Neg |
| [[13]](https://paperpile.com/c/d5ULsP/kLop) | 17 | M | France | France | M | NR | 840 (L) | Normal, NR | Normal, NR | IgM+/IgG+ | Pos | NT |
| [[14]](https://paperpile.com/c/d5ULsP/N6yP) | 34 | F | Italy | Italy | M | NR | 320 (L) | 45 | 101 | One serum sample tested positive for IgM TOSV, all the other serum samples tested positive for both IgG and IgM TOSV | 11 CSF samples tested positive for RT-PCR TOSV | NT |
|  | 18 | F | Italy | Italy | M | NR | 880 (L) | 49 | 130 |  |  | NT |
|  | 74 | M | Italy | Italy | ME | NR | 248 (L) | 40 | 137 |  |  | NT |
|  | 50 | M | Italy | Italy | M | NR | 992 (L) | 65 | 184 |  |  | NT |
|  | 28 | M | Italy | Italy | M | NR | 1255 (L) | 79 | 144 |  |  | NT |
|  | 25 | M | Italy | Italy | M | NR | 464 (L) | 68 | 53 |  |  | NT |
|  | 36 | F | Italy | Italy | M | NR | 371 (L) | 51 | 148 |  |  | NT |
|  | 74 | F | Italy | Italy | ME | NR | 160 (L) | 50 | 117 |  |  | NT |
|  | 42 | M | Italy | Italy | M | NR | 180 (L) | 58 | 158 |  |  | NT |
|  | 36 | M | Italy | Italy | M | NR | 176 (L) | 52 | 173 |  |  | NT |
|  | 45 | M | Italy | Italy | M | NR | 660 (L) | 73 | 180 |  |  | NT |
|  | 17 | M | Italy | Italy | M | NR | 40 (L) | 57 | 62 |  |  | NT |
|  | 34 | M | Italy | Italy | M | NR | 375 (L) | 43 | 181 |  |  | NT |
|  | 27 | F | Italy | Italy | M | NR | 364 (L) | 59 | 78 |  |  | NT |
|  | 48 | F | Italy | Italy | M | NR | 156 (L) | 45 | 70 |  |  | NT |
|  | 37 | M | Italy | Italy | M | NR | 420 (L) | 59 | 220 |  |  | NT |
| [[15]](https://paperpile.com/c/d5ULsP/f25w) | 32 | M | Italy | Italy | M | Slightly turbid | 95 (L) | 43 | 139 | IgM+/IgG+ | Pos | NT |
| [[16]](https://paperpile.com/c/d5ULsP/zc4J) | 8 | M | Greece | Greece | ME | NR | 1 (NR) | 64 | 26 | IgM-/IgG+ | NT | Neg |
| [[17]](https://paperpile.com/c/d5ULsP/n5tr) | 49 | M | Italy | Italy | E | Clear | 35 (NR) | Normal, NR | 77 | IgM-/IgG+ | NT | NT |
| [[18]](https://paperpile.com/c/d5ULsP/CWBm) | 76 | M | France | France | E | NR | 8 (NR) | 59 | 87 | IgM+/IgG- | NT | NT |
| [[19]](https://paperpile.com/c/d5ULsP/uY3K) | 45 | M | Spain | Spain | ME | NR | Elevated (NR, L) | Normal, NR | Elevated, NR | IgM+/IgG NR | NT | NT |
|  | 54 | M | Spain | Spain | ME | NR | Elevated (NR, L) | Normal, NR | Elevated, NR | IgM+/IgG+ | NT | NT |
| [[20]](https://paperpile.com/c/d5ULsP/BZuH) | 65 | M | Italy | USA | E | NR | 14 (L) | 63 | 126 | IgM+/IgG NR | NT | NT |
| [[21]](https://paperpile.com/c/d5ULsP/ZNjB) | 20 | M | Italy | Switzerland | M | Clear | 196 (Mo) | 67 | 75 | IgM+/IgG+ | Neg | NT |
| [[22]](https://paperpile.com/c/d5ULsP/X1h5) | 68 | M | Italy | France | ME | NR | 791 (L) | 40 | 202 | IgM+/IgG+ | NT | NT |
| [[23]](https://paperpile.com/c/d5ULsP/SpOq) | 63 | M | Spain | Spain | M | Clear | 70 (P) | 61 | 81 | IgM+/IgG+ | Pos | NT |
| [[24]](https://paperpile.com/c/d5ULsP/SDko) | 80 | M | Spain | Germany | ME | NR | 265 (NR) | NR | 47 | IgM+/IgG+ | NT | NT |
| [[25]](https://paperpile.com/c/d5ULsP/AQ0g) | 49 | F | Italy | France | M | NR | 175 (L) | 58 | 87 | IgM+/IgG+ | NT | NT |
| [[26]](https://paperpile.com/c/d5ULsP/0hgE) | 57 | F | France | France | M | NR | 3500 (L) | 45 | 274.9 | IgM+/IgG+ | NT | NT |
| [[27]](https://paperpile.com/c/d5ULsP/JMxf) | 19 | F | Italy | Italy | ME | Clear | 300 (Mo) | 106 | 280 | IgM+/IgG+ | Pos | NT |
|  | 16 | M | Italy | Italy | ME | Clear | 200 (Mo) | 79 | 180 | IgM+/IgG+ | Pos | NT |
| [[28]](https://paperpile.com/c/d5ULsP/MT0G) | Children < 14 years of ages | | Italy | Italy | NR | NR | 45 (NR) | 100 | 30 | IgM+/IgG+ | NT | NT |
|  |  |  | Italy | Italy | NR | NR | 300 (NR) | 45 | 45 | IgM+/IgG+ | NT | NT |
|  |  |  | Italy | Italy | NR | NR | 260 (NR) | 29 | 40 | IgM+/IgG+ | NT | NT |
|  |  |  | Italy | Italy | NR | NR | 300 (NR) | 45 | 25 | IgM+/IgG+ | NT | NT |
|  |  |  | Italy | Italy | NR | NR | 35 (NR) | 40 | 30 | IgM+/IgG+ | NT | NT |
|  |  |  | Italy | Italy | NR | NR | 38 (NR) | 58 | 100 | IgM+/IgG+ | NT | NT |
|  |  |  | Italy | Italy | NR | NR | 350 (NR) | 38 | 45 | IgM+/IgG+ | NT | NT |
|  |  |  | Italy | Italy | NR | NR | 1200 (NR) | 90 | 70 | IgM+/IgG+ | NT | NT |
|  |  |  | Italy | Italy | NR | NR | 220 (NR) | 57 | 40 | IgM+/IgG+ | NT | NT |
|  |  |  | Italy | Italy | NR | NR | 30 (NR) | 78 | 20 | IgM+/IgG+ | NT | NT |
|  |  |  | Italy | Italy | NR | NR | 70 (NR) | 56 | 40 | IgM+/IgG+ | NT | NT |
|  |  |  | Italy | Italy | NR | NR | 220 (NR) | 64 | 35 | IgM+/IgG+ | NT | NT |
|  |  |  | Italy | Italy | NR | NR | 220 (NR) | 64 | 35 | IgM+/IgG+ | NT | NT |
|  |  |  | Italy | Italy | NR | NR | 650 (NR) | 46 | 89 | IgM+/IgG+ | NT | NT |
| [[29]](https://paperpile.com/c/d5ULsP/FwJ0) | 53 | M | Italy | Germany | M | NR | 408 (L) | 78 | 184 | IgM+/IgG+ | NT | NT |
| [[30]](https://paperpile.com/c/d5ULsP/xNYd) | 66 | M | Italy | USA | E | NR | 710 (Mo) | 66 | 180 | IgM+/IgG NR | NT | NT |

**References**

[1] Tschumi F, Schmutz S, Kufner V, Heider M, Pigny F, Schreiner, et al. Meningitis and epididymitis caused by Toscana virus infection imported to Switzerland diagnosed by metagenomic sequencing: a case report. BMC Infect Dis 2019;19(1):591. https://doi.org/10.1186/s12879-019-4231-9.

[2] Vilibic-Cavlek T, Zidovec-Lepej S, Ledina D, Knezevic S, Savic V, Tabain I, et al. Clinical, Virological, and Immunological Findings in Patients with Toscana Neuroinvasive Disease in Croatia: Report of Three Cases. Trop Med Infect Dis 2020;5(3):144. https://doi.org/10.3390/tropicalmed5030144.

[3] Suardi LR, Di Lauria N, Pozzi M, Rogasi PG, Barilaro A, Azzolini F, et al. Acute cerebellar ataxia: a rare Toscana Virus (TOSV) meningoencephalitis complication. Int J Neurosci 2020;130(3):276-278. https://doi.org/10.1080/00207454.2019.1673748.

[4] Gharsallah H, Tritar A, Naija H, Batikh R, Ferjani M. Severe meningoencephalomyelitis due to toscana virus: A diagnostic challenge. Travel Med Infect Dis 2021;43:102131. https://doi.org/10.1016/j.tmaid.2021.102131.

[5] Mascitti H, Calin R, Dinh A, Makhloufi S, Davido B. Testicular pain associated with clear fluid meningitis: How many cases of Toscana virus are we missing? Int J Infect Dis 2020;93:198-200. https://doi.org/10.1016/j.ijid.2020.02.008.

[6] Karunaratne K, Davies N. Toscana virus meningitis following a holiday in Elba, Italy. Br J Hosp Med 2018;79(5):292. https://doi.org/10.12968/hmed.2018.79.5.292.

[7] Arden KE, Heney C, Shaban B, Nimmo GR, Nissen MD, Sloots TP, et al. Detection of Toscana virus from an adult traveler returning to Australia with encephalitis. J Med Virol 2017;89(10):1861-1864. https://doi.org/10.1002/jmv.24839.

[8] Osborne JC, Khatamzas E, Misbahuddin A, Hart R, Sivaramakrishnan A, Breen DP. Toscana virus encephalitis following a holiday in Sicily. Pract Neurol 2016;16(2):139-41. https://doi.org/10.1136/practneurol-2015-001265.

[9] Howell BA, Azar MM, Landry ML, Shaw AC. Toscana virus encephalitis in a traveler returning to the United States. J Clin Microbiol 2015;53(4):1445-7. https://doi.org/10.1128/JCM.03498-14.

[10] Cordey S, Bel M, Petty TJ, Docquier M, Sacco L, Turin L, et al. Toscana virus meningitis case in Switzerland: an example of the ezVIR bioinformatics pipeline utility for the identification of emerging viruses. Clin Microbiol Infect 2015;21(4):387.e1-4. https://doi.org/10.1016/j.cmi.2014.11.010.

[11] Marlinge M, Crespy L, Zandotti C, Piorkowski G, Kaphan E, Charrel RN, et al. Afebrile meningoencephalitis with transient central facial paralysis due to Toscana virus infection, southeastern France, 2014. Euro Surveill 2014;19(48):20974. https://doi.org/10.2807/1560-7917.es2014.19.48.20974.

[12] Magurano F, Baggieri M, Gattuso G, Fortuna C, Remoli ME, Vaccari G, et al. Toscana virus genome stability: data from a meningoencephalitis case in Mantua, Italy. Vector Borne Zoonotic Dis 2014;14(12):866-9. https://doi.org/10.1089/vbz.2014.1668.

[13] Nougairede A, Bichaud L, Thiberville S-D, Ninove L, Zandotti C, de Lamballerie X, et al. Isolation of Toscana virus from the cerebrospinal fluid of a man with meningitis in Marseille, France, 2010. Vector Borne Zoonotic Dis 2013;13(9):685-8. https://doi.org/10.1089/vbz.2013.1316.

[14] Vocale C, Bartoletti M, Rossini G, Macini P, Pascucci MG, Mori F, et al. Toscana virus infections in northern Italy: laboratory and clinical evaluation. Vector Borne Zoonotic Dis 2012;12(6):526-9. https://doi.org/10.1089/vbz.2011.0781.

[15] [Greco F, Mauro MV, Tenuta R, Apuzzo G, Giraldi C. A new case of meningitis due to Toscana virus. New Microbiol 2012;35(1):99–100](http://paperpile.com/b/d5ULsP/f25w).

[16] Anagnostou V, Sdouga M, Volakli H, Violaki A, Papa A. Phlebovirus meningoencephalis complicated by Pseudomonas aeruginosa pneumonia: a case report. Vector Borne Zoonotic Dis 2011;11(5):595-6. https://doi.org/10.1089/vbz.2010.0041.

[17] Serata D, Rapinesi C, Del Casale A, Simonetti A, Mazzarini L, Ambrosi E, et al. Personality changes after Toscana virus (TOSV) encephalitis in a 49-year-old man: A case report. Int J Neurosci 2011;121(3):165-9. https://doi.org/10.3109/00207454.2010.537412.

[18] Doudier B, Ninove L, Million M, de Lamballerie X, Charrel R-N, Brouqui P. Unusual Toscana virus encephalitis in southern France. Med Mal Infect 2011;41(1):50-1. https://doi.org/10.1016/j.medmal.2010.09.006.

[19] Sanbonmatsu-Gámez S, Pérez-Ruiz M, Palop-Borrás B, Navarro-Marí JM. Unusual manifestation of toscana virus infection, Spain. Emerg Infect Dis 2009;15(2):347-8. https://doi.org/10.3201/eid1502.081001.

[20] Kay MK, Gibney KB, Riedo FX, Kosoy OL, Lanciotti RS, Lambert AJ. Toscana virus infection in American traveler returning from Sicily, 2009. Emerg Infect Dis 2010;16(9):1498-500. https://doi.org/10.3201/eid1609.100505.

[21] [Sonderegger B, Hachler H, Dobler G, Frei M. Imported aseptic meningitis due to Toscana virus acquired on the island of Elba, Italy, August 2008. Euro Surveill 2009;14(1):19079](http://paperpile.com/b/d5ULsP/ZNjB).

[22] Epelboin L, Hausfater P, Schuffenecker I, Riou B, Zeller H, Bricaire F, et al. Meningoencephalitis due to Toscana virus in a French traveler returning from central Italy. J Travel Med 2008;15(5):361-3. https://doi.org/10.1111/j.1708-8305.2008.00221.x.

[23] Martínez-García FA, Moreno-Docón A, Segovia-Hernández M, Fernández-Barreiro A. Deafness as a sequela of Toscana virus meningitis. Med Clin 2008;130(16):639. https://doi.org/10.1157/13120347.

[24] Kuhn J, Bewermeyer H, Hartmann-Klosterkoetter U, Emmerich P, Schilling S, Valassina M. Toscana virus causing severe meningoencephalitis in an elderly traveller. J Neurol Neurosurg Psychiatry 2005;76(11):1605-6. https://doi.org/10.1136/jnnp.2004.060863.

[25] Defuentes G, Rapp C, Imbert P, Durand J-P, Debord T. Acute meningitis owing to phlebotomus fever Toscana virus imported to France. J Travel Med 2005;12(5):295-6. https://doi.org/10.2310/7060.2005.12512.

[26] Peyrefitte CN, Devetakov I, Pastorino B, Villeneuve L, Bessaud M, Stolidi P, et al. Toscana virus and acute meningitis, France. Emerg Infect Dis 2005;11(5):778-80. https://doi.org/10.3201/eid1105.041122.

[27] Baldelli F, Ciufolini MG, Francisci D, Marchi A, Venturi G, Fiorentini C, et al. Unusual presentation of life-threatening Toscana virus meningoencephalitis. Clin Infect Dis 2004;38(4):515-20. https://doi.org/10.1086/381201.

[28] Braito A, Corbisiero R, Corradini S, Fiorentini C, Ciufolini MG. Toscana virus infections of the central nervous system in children: a report of 14 cases. J Pediatr 1998;132(1):144-8. https://doi.org/10.1016/s0022-3476(98)70500-1.

[29] Schwarz TF, Gilch S, Jäger G. Travel-related Toscana virus infection. Lancet 1993;342(8874):803-4. https://doi.org/10.1016/0140-6736(93)91568-7.

[30] Calisher CH, Weinberg AN, Muth DJ, Lazuick JS. Toscana virus infection in United States citizen returning from Italy. Lancet 1987;1(8525):165-6. https://doi.org/10.1016/s0140-6736(87)92005-8.
